# Whole-Genome Sequencing and Annotation of the Yeast *Clavispora santaluciae* Reveals Important Insights about Its Adaptation to the Vineyard Environment

**DOI:** 10.3390/jof8010052

**Published:** 2022-01-05

**Authors:** Ricardo Franco-Duarte, Neža Čadež, Teresa Rito, João Drumonde-Neves, Yazmid Reyes Dominguez, Célia Pais, Maria João Sousa, Pedro Soares

**Affiliations:** 1 CBMA, Centre of Molecular and Environmental Biology, Department of Biology, University of Minho, 4710-057 Braga, Portugal; teresarito@bio.uminho.pt (T.R.); cpais@bio.uminho.pt (C.P.); mjsousa@bio.uminho.pt (M.J.S.); pedrosoares@bio.uminho.pt (P.S.); 2 Institute of Science and Innovation for Bio-Sustainability (IB-S), University of Minho, 4710-057 Braga, Portugal; 3 Department of Food Science and Technology, Biotechnical Faculty, University of Ljubljana, 101, 1000 Ljubljana, Slovenia; neza.cadez@bf.uni-lj.si; 4 IITAA—Institute of Agricultural and Environmental Research and Technology, University of Azores, 9700-042 Angra do Heroísmo, Portugal; drumondeneves@gmail.com; 5 Laimburg Research Centre, Laimburg 6, 39052 Vadena, Italy; Yazmid.Reyes-Dominguez@laimburg.it

**Keywords:** genomics, phylogenomics, functional gene analysis, Metschnikowiaceae, Azores, wine yeasts, biotechnology, adaptation

## Abstract

*Clavispora santaluciae* was recently described as a novel non-*Saccharomyces* yeast species, isolated from grapes of Azores vineyards, a Portuguese archipelago with particular environmental conditions, and from Italian grapes infected with *Drosophila suzukii*. In the present work, the genome of five *Clavispora santaluciae* strains was sequenced, assembled, and annotated for the first time, using robust pipelines, and a combination of both long- and short-read sequencing platforms. Genome comparisons revealed specific differences between strains of *Clavispora santaluciae* reflecting their isolation in two separate ecological niches—Azorean and Italian vineyards—as well as mechanisms of adaptation to the intricate and arduous environmental features of the geographical location from which they were isolated. In particular, relevant differences were detected in the number of coding genes (shared and unique) and transposable elements, the amount and diversity of non-coding RNAs, and the enzymatic potential of each strain through the analysis of their CAZyome. A comparative study was also conducted between the *Clavispora santaluciae* genome and those of the remaining species of the Metschnikowiaceae family. Our phylogenetic and genomic analysis, comprising 126 yeast strains (alignment of 2362 common proteins) allowed the establishment of a robust phylogram of Metschnikowiaceae and detailed incongruencies to be clarified in the future.

## 1. Introduction

In our previous surveys on the yeast diversity of Azorean vineyards, in 2009 and 2010 [1,2,3], we described a new yeast species *Clavispora santaluciae* [4], isolated from grapes. It was characterized on the basis of the sequences of the internal transcribed spacer (ITS) region (ITS1-5.8S–ITS2), the sequences of the D1/D2 domain of the large subunit (LSU) rRNA gene, and particular physiological characteristics. That study also described this species as being isolated from grapes infected with *Drosophila suzukii* in Italy. This showed identical D1/D2 sequences and very similar ITS regions (five nucleotide substitutions) to the Azorean strains. The new species was obtained from particular viticultural environments, typical of the Azores archipelago, which result from the interaction between specific climatic conditions, autochthonous grapevine cultivars, and local viticultural practices. Phenotypic characterization of this new species revealed some interesting features that positioned it apart from the closely related ones, such as the inability to grow at temperatures above 35 °C, production of acetic acid, and the capacity to assimilate starch. The full biotechnological potential of this new species remains to be explored, as does the understanding of the genomic features associated with the adaptation to its environment. The occurrence of biotechnologically important features associated with species of this clade is not uncommon. *(Candida*) *intermedia*, a xylose-utilizing species of the Metschnikowiaceae family, belonging to the *Clavispora* clade, displays a high-capacity xylose transport system [5,6]. Due to these characteristics, this species has been categorized as an attractive species to produce ethanol from lignocellulosic biomass [7].

Our previous study was one of the few to report the presence of yeast species belonging to the *Clavispora* clade in vineyards, even though some rare reports have already associated these yeasts with winemaking, as detailed below. We highlighted the rarity of these occurrences, in a review of the association of non-*Saccharomyces* yeasts with viticulture and winemaking [8]. In that study, we systematized 80 years of the literature describing non-*Saccharomyces* yeast species isolated from grapes and/or grape musts and compiled a list of 293 species. Only two species belonging to the *Clavispora* clade were identified—*Clavispora fructus* and *Clavispora lusitaniae*. Even though there is no strong association between *Clavispora* species and wine, some reports have already described the possible advantages of these yeasts in the production of wines with alternative sensory characteristics, albeit with some associated disadvantages, such as the presence of an abnormally high concentration of acetaldehyde [9]. In Azorean vineyards, no other yeast of this genus was found within the 2910 isolates identified in our previous work [1,2], but a single species of this family (*Metschnikowia pulcherrima)* was found in four different islands in two consecutive sampling years. The use of this species in wine biotechnology was recently reviewed [10], with the authors concluding that its versatility lies in its ability to ferment must in combination with other yeast species (mainly to circumvent its low fermentative power), as well as modulating the synthesis of secondary fermentation metabolites to improve and diversify the sensory profile of the wine.

The phylogram obtained in our previous study [4], based on concatenated sequences of the D1/D2 domain of the LSU rRNA gene, and of the ITS region, placed *Clavispora santaluciae* strains near closely the related species *Clavispora fructus*, (*Candida*) *asparagi*, (*Candida*) *vitiphila*, (*Candida*) *phyllophila*, (*Candida*) *carvajalis*, and *Clavispora lusitaniae*. The description of this new species was considered as an important add-on in understanding the phylogenetic relationships within the *Clavispora* clade and clarifying their biodiversity and ecology. Previously, several yeasts that now belong to the *Clavispora* genus, were placed in the anamorphic genus *Candida*, due to their inability to form sexual spores [11]. With recent phylogenetic analysis based on DNA sequences, in combination with physiological evidence, the relationships between the species of *Candida* and the genus *Clavispora* began to be clarified, leading to a new classification of this group by Daniel et al. in 2014 [11], with 40 species of *Candida* assigned to the genus *Clavispora*, based on sequences of the LSU D1/D2 domain, ITS region and four coding genes (*ACT1*, *TEF1*, *MCM7,* and *RPB2*). In 2018, Kurtzman et al. [12] described four new species of *Metschnikowia* and proposed to transfer seven additional *Candida* species to both *Metschnikowia* and *Clavispora* genera. Kurtzman et al. concluded that the taxonomy of the *Clavispora* clade could only be clarified by whole-genome comparisons, which still need to be performed. Regarding the *Clavispora* genus, only two species have been genome sequenced and annotated: *Clavispora lusitaniae* (11.9–12.1 Mb, 8 chromosomes, genome accession ASM167369v2 ), and *Clavispora fructus* (11.4 Mb, NCBI genome accession ASM370779v1). In 2016, a phylogeny of the Metschnikowiaceae family was presented by Lachance et al. [13], combining draft genomes of 55 strains and identifying 3016 orthologues, 1061 of which exist in all the analyzed strains. Even though the *Clavispora lusitaniae* genome was used as a query to compare between all the genomes, no species belonging to the *Clavispora* genus were considered, with the analysis focused on only the *Metschnikowia* genus. More recently, Shen et al. [14] attempted to reconstruct the phylogeny of 300 budding yeast species, focusing mainly on the diversity of Saccharomycotina. In that work, whole genomes were used to partially describe the phylogeny of Metschnikowiaceae clade considering only 22 *Metschnikowia* species. This analysis used only the type strains of each species, which lack intra-species diversity, and the genome annotation was not directed to the analysis of this clade. Thus, a deep, broad, and focused analysis using robustly annotated genomes of the phylogenetic relations between *Clavispora* species (including also the recently assigned *Candida* species) and the sister genus *Metschnikowia* is lacking. With this in mind, the objective of the present work was to sequence, assemble and annotate whole genomes of the different *Clavispora santaluciae* strains, using a combination of both long- and short-read sequencing platforms, in order to obtain high-quality sequences for comparative genomics. We used the assembled genomes to further elucidate the molecular mechanisms underlying the adaptation of this species to the particular environmental characteristics from which it was isolated. In particular, the main goal was to unravel the genomic features that can explain the phenotypic characteristics previously observed within isolates of *Clavispora santaluciae*, as well as to predict their biotechnological potential. In addition, all species from the Metschnikowiaceae family whose complete genome was publicly available were considered and combined in phylogenetic and genomic analysis, to help clarify the phylogenetic placement of *Clavispora* yeasts within this large group of important non-*Saccharomyces* yeasts. We plan to use our results to clarify the positioning of (*Candida*) species in relation to the sister genus *Metschnikowia* and reconstruct Metschnikowiaceae family phylogeny using complete genomes.

## 2. Materials and Methods

### 2.1. Cell Culture, Sample Collection, and DNA Extraction

The type strain of *Clavispora santaluciae* (A1.18^T^ = CBS 16465^T^), together with the three strains isolated from grapes of Azorean vineyards (A1.5, A1.7, and A1.19), and one additional strain isolated from grapes infected with *Drosophila suzukii* in Italy (LB-NB-3.3) [4], were grown in YPD broth (yeast extract, 1% *w/v*; peptone, 1% *w/v*; glucose 2% *w/v*), in 50 mL conical flasks, for 48 h at 28 °C, 220 rpm. Genomic DNA was isolated according to the protocol published by Schwartz and Sherlock [15], with a few adaptations for the isolation of DNA from non-*Saccharomyces* yeasts. After washing in 0.9 M sorbitol solution, the cells were incubated by adding 20 μL of Lyticase (30 mg/mL, Sigma-Aldrich, St. Louis, MO, USA) to the cells. The incubation time was at least 4 h at 37 °C. Following phenol/chloroform (Millipore, Burlington, MA, USA) extraction, the DNA was precipitated using 40 μL of 3 M sodium acetate (pH 5.5) and 1 mL of absolute ethanol and resuspended in 200 μL of TE buffer.

### 2.2. Genome Sequencing and Assembly

The genomes of all the *Clavispora santaluciae* strains were sequenced by using a combination of the long-and short-read sequencing technologies of PacBio and Illumina, respectively. After DNA extraction, library preparation and PacBio/Illumina sequencing were performed at Novogene facilities (Novogene Company LTD, Cambridge, United Kingdom). Low-quality reads and adapters were removed by Novogene, and sequencing quality was accessed using FastQC software (http://www.bioinformatics.bbsrc.ac.uk/projects/fastqc/; accessed on 1 August 2021). The sequencing data are available at NCBI BioProject ID PRJNA784374.

Long-reads obtained from PacBio sequencing were de novo assembled using Canu v.1.9 [16] with default parameters. Illumina paired-end reads were then used to improve assembly quality, using Masurca software v.4.0.5, in particular the Polca package [17,18]. Finally, RagTag software v.2.1.0 [19] was used to assemble all the scaffolds into longer reads, using chromosome information from the closely related species *Clavispora lusitaniae* and (*Candida*) *intermedia*. Genome assembly quality metrics, available in Table 1, were computed using QUAST v.5.0.2 [20].

To determine ploidy, we used nQuire software [21] to align sequencing reads to the type strain genome assembled after RagTag and determine base frequency distributions between frequencies 20 and 80. Assessment of each genomes’ completeness was performed using Benchmarking Universal Single-Copy Orthologs (BUSCO) software v.5.2.2 [22]. Average nucleotide identity (ANI) was calculated using the OrthoANIu web tool [23] in pairwise mode, to compare the nucleotide content of genomes.

### 2.3. Genome Annotation

Annotation of *Clavispora santaluciae* genome assemblies was performed using AUGUSTUS software v.3.4.0 [24,25], considering 11 different pre-trained models, chosen as belonging to the Ascomycota phyla *Saccharomyces cerevisiae* S288c, *Candida albicans, Meyerozyma* (*Candida*) *guilliermondii, Candida tropicalis, Debaryomyces hansenii, Eremothecium gossypii, Kluyveromyces lactis, Lodderomyces elongisporus, Scheffersomyces (Pichia) stipitis, Schizosaccharomyces pombe,* and *Yarrowia lipolytica.* Results were manually reviewed to select the annotation with the higher number of predicted coding genes, which was obtained using *Lodderomyces elongisporus* as the pre-trained model, for all the *Clavispora santaluciae* strains. The potential coding regions (nucleotide sequences) reported by AUGUSTUS were extracted from the complete genomes to FASTA files.

CMsearch [26] and StructRNAfinder [27] were used for screening the presence of non-coding RNA (ncRNA). The Rfam database [28] was employed for ncRNA searching, using an e-value of 0.01.

Functional genomic annotation was performed with eggNOG-mapper v.2 [29] by considering proteins predicted by AUGUSTUS and choosing only orthologs that were inferred from the experimental evidence. The results were described considering clusters of orthologous groups (COGs) with their associated functional categories [30], and also considering the Kyoto Encyclopedia of Genes and Genomes (KEGG) pathways, in particular the KEGG Orthology (KO) descriptors [31,32]. Gene function predictions were also accomplished by assessing the Carbohydrate-Active EnZymes (CAZymes) database [33].

Final genome annotations for all *Clavispora santaluciae* strains are available in Supplementary Data S1.

### 2.4. Homology Analysis, Comparative Genomics, and Phylogenomics

To compare the genome of *Clavispora santaluciae* type strain A1.18 with that of the remaining strains, dot plots were produced using the Re-Dot-Able tool (https://www.bioinformatics.babraham.ac.uk/projects/redotable/). Inter-species differences between members of the family Metschnikowiaceae were evaluated by downloading all the complete genomes publicly available at NCBI (121 strains belonging to 48 different species). When more than one strain was available for a certain species, all strains were considered. The exception was (*Candida*) *auris* for which only the representative genome was used since the hundreds of strains with genome sequence available would have increased redundancy.

KEGG Mapper was used as a collection of KEGG mapping tools for linking genes and proteins to metabolic pathways [32,34]. In particular, KO gene annotations, obtained from eggnog-mapper, were used to assess pathway completeness using KEGG Mapper–Reconstruct web tool (www.genome.jp/kegg/mapper/reconstruct.html). Results were applied in the construction of a heatmap using Microsoft Excel^®^. 

A database was prepared by considering all 126 complete genomes (121 strains of Metschnikowiaceae family plus the 5 *Clavispora santaluciae* isolates). To avoid inconsistency, the 121 Metschnikowiaceae genomes were annotated using Augustus with the same pre-trained model as was applied for the annotation of the *Clavispora santaluciae* genomes. BLASTP analysis was performed using the full proteome of the *Clavispora santaluciae* type strain A1.18^T^ as a query against the total database. An *E*-value cutoff of 10^−6^ was used to exclude false results, and a pipeline adapted from [35] was used to perform comparative genomics between all isolates. The BLASTP results were filtered where representative proteins were detected in the other 121 isolates. Each set of probable homologous proteins (containing the query and the respective results) were multiple aligned using the MAFFT algorithm in FasParser (https://github.com/Sun-Yanbo/FasParser) [36]. All proteins from a given organism were concatenated using the alignment results to obtain the core conserved aligned proteome containing mostly essential genes not related to specific biological traits of each species. This alignment was then used for phylogenetic reconstruction by considering the maximum likelihood in IQ-TREE (www.iqtree.org) [37], with the JTT model of amino acid evolution and gamma-distributed rates (four rates) with 500 bootstrap replicates. Two outgroups were considered: *Lipomyces lipofer* and *Cyberlindnera jadinii*. FigTree v.1.4.4 (http://tree.bio.ed.ac.uk/software/figtree/) was used to visualize and edit the tree. The second round of BLASTP analysis, using the proteomes of the five *Clavispora santaluciae* strains as queries, allowed building Venn diagrams to schematize the number of genes common between the five genomes using the average results between all pairs or between groups of three, four, or in all five strains.

## 3. Results and Discussion

### 3.1. Sequencing, De Novo Assembly, and Annotation of Clavispora Santaluciae Genome

Genome sequencing of *Clavispora santaluciae* strains A1.18^T^, A1.5, A1.7, A1.19, and LB-NB-3.3 was performed using a combination of long- and short-read sequencing platforms. Between 27,903 and 44,977 reads were obtained with long-read sequencing, with a maximum read length of 110,418 base pairs (bp). Short-read sequencing was used to refine long-read sequencing results. An average value of 3 × 10^6^ paired-end reads, with 250 bp each, was obtained for each strain. The first round of assembly was performed using Canu and Masurca assemblers (sequencing statistics are presented in Table 1), and then RagTag software assembled the scaffolds into putative chromosomes. By using three assemblers we were able to assemble long and short-read sequences into full chromosomes for three of the strains, including the type strain A1.18^T^ and strains A1.5 and LB-NB-3.3. The remaining two strains, possibly due to lower sequencing depth, were only assembled into large scaffolds. The attained haploid genome size (10.8 Mb to 11.1 Mb) was comparable with the previously published genomes of *Clavispora* yeasts, in particular with the 11.9–12.1 Mb of *Clavispora lusitaniae* (8 chromosomes) [38,39], or with the 11.4 Mb of *Clavispora fructus* (NCBI genome accession ASM370779v1).

The high-quality-assembled genomes allowed the prediction of between 6015 and 6092 protein-coding genes for the five *Clavispora lusitaniae* strains using AUGUSTUS software (Table 2, Supplementary Data S1). These values are among the highest reported for yeasts of the *Clavispora* clade, and are comparable only to the annotation of one ((*Candida*) *intermedia* strain YCC 4715), for which 6082 coding genes were predicted [40], but corresponding to a greater genome length of 13.08Mb.

The unusually high number of predicted proteins in the genome of *Clavispora santaluciae* was likely not related, in our opinion, to any peculiarity of this yeast´s genome but rather to the use of advanced sequencing technologies, together with an improved annotation pipeline. The lowest number of predicted coding sequences was determined for strain A1.19. This could be attributed to lower sequencing depth. This was also the shortest genome of the five, the one with the lowest N50 values (Table 1), and the one with lower BUSCO genome completeness scores, both in Ascomycota and Saccharomycetes databases (Table 2).

The highest number of predicted proteins was described in the annotation of the genome of the type strain A1.18^T^, with 6092 coding sequences (Table 2). The average length of the predicted proteins was slightly lower in LB-NB-3.3, although the largest protein of 5293 amino acids (aa) was annotated in this strain. This large open reading frame encodes the protein midasin (Mdn1), an ATPase of 560 kDa that is essential for cell viability. It was identified in all *Clavispora santaluciae* strains and reported in other yeasts, such as in the genera *Saccharomyces* and *Schizosaccharomyces,* as well as in distant organisms as *Drosophila* and *Arabidopsis* [41]. The lowest coding sequence annotated (57 aa) corresponds to a hypothetical protein not yet characterized in the Metschnikowiaceae (data not shown) but identified as a mitochondrial ATP synthase ε chain-domain-containing protein in the *Terfezia claveryi* mycorrhizal fungus (NCBI accession KAF8454923.1). The fact that we found no proteins below this size, which could correspond to the annotation of false positives, highlights the high annotation quality obtained with the computational pipeline and the sequencing technology applied.

The total number of non-coding RNAs (ncRNA) predicted using structRNAfinder and the Pfam database was similar among the five *Clavispora santaluciae* strains (Table 2, Supplementary Data S2). There was a high similarity between strains for the majority of the ncRNA annotated, with the exception of ribosomal and transfer RNAs (rRNA and tRNA, respectively), whose quantities showed relevant inter-strain variation not directly correlated with the number of predicted coding sequences or with the genome size. Many sequencing projects ignore the comparison of ncRNA between strains, but by detailing their analysis, it may be possible to understand particular and intricate mechanisms of adaptation to the environment.

### 3.2. Comparative Genomics of Clavispora Santalucieae Strains

**Figure 1:**
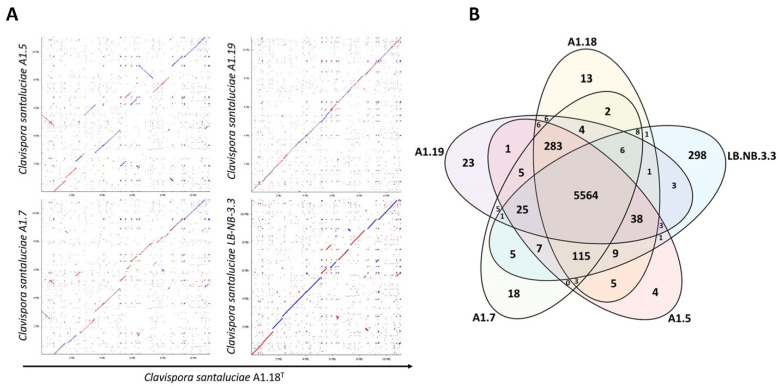
Comparative genomics of *Clavispora santaluciae* genomes: (**A**) whole-genome dot-plot comparison between the sequenced strains in pairwise mode. Homologous regions are plotted as dots. Red lines link parallel homologous pairs, and blue lines link anti-parallel pairs; (**B**) Venn diagram indicating the number of shared coding genes among *Clavispora santaluciae* strains.

To compare structural variations between the genomes of the *Clavispora santaluciae* strains pairwise, dot plots were obtained (Figure 1A). Results showed a striking pattern of conservation for most strains, with a high degree of macrosynteny mainly between the type strain and strains A1.19 and LB-NB-3.3. On the other hand, strains A1.5 and A1.7 showed some differentiation, in particular by the presence of several deletions in parts of the genome, as represented by translocations (“jumps” in the dot plot) away from the main diagonal. In particular, strain A1.5 seems to have mesosynteny with the type strain, since we can generally observe conservation of the gene content. However, in some parts of the genome, many inversions (blue lines) and translocations were detected. This observation is not concordant with the similarities observed in the ITS and D1/D2 regions [4], which showed that strain A1.5 is most closely related to the type strain.

A total of 5564 coding genes were found to be shared between the five *Clavispora santaluciae* strains, corresponding to the pangenome of the species (Figure 1B). Strain NB-LB-3.3 showed a surprisingly high number of unique genes (298), not shared by any of the other strains, reflecting its adaptation to a different ecological niche, as this strain was isolated from Italian grapes infected with *Drosophila suzukii*. On the other hand, 283 genes were shared only by the strains isolated from Azorean vineyards, indicating adaptation mechanisms to the intricate and arduous environmental conditions of the geographical location from which they were isolated. Additionally, and of particular note, is the fact that no transposable element was identified in the genome of strain LB-NB-3.3, unlike the other four Azorean strains (Supplementary Data S1). According to our previous work [42] on the characterization of isogenic isolates of wine *S. cerevisiae* yeasts, transposable elements seem to be related to the adaptation of yeasts to the fluctuating environmental conditions found in the harsh environment of the Azores archipelago, and these genetic features are related with important phenotypic characteristics that determine the strains biotechnological potential [43,44].

### 3.3. Functional Annotation of Clavispora Santaluciae Proteome

**Figure 2:**
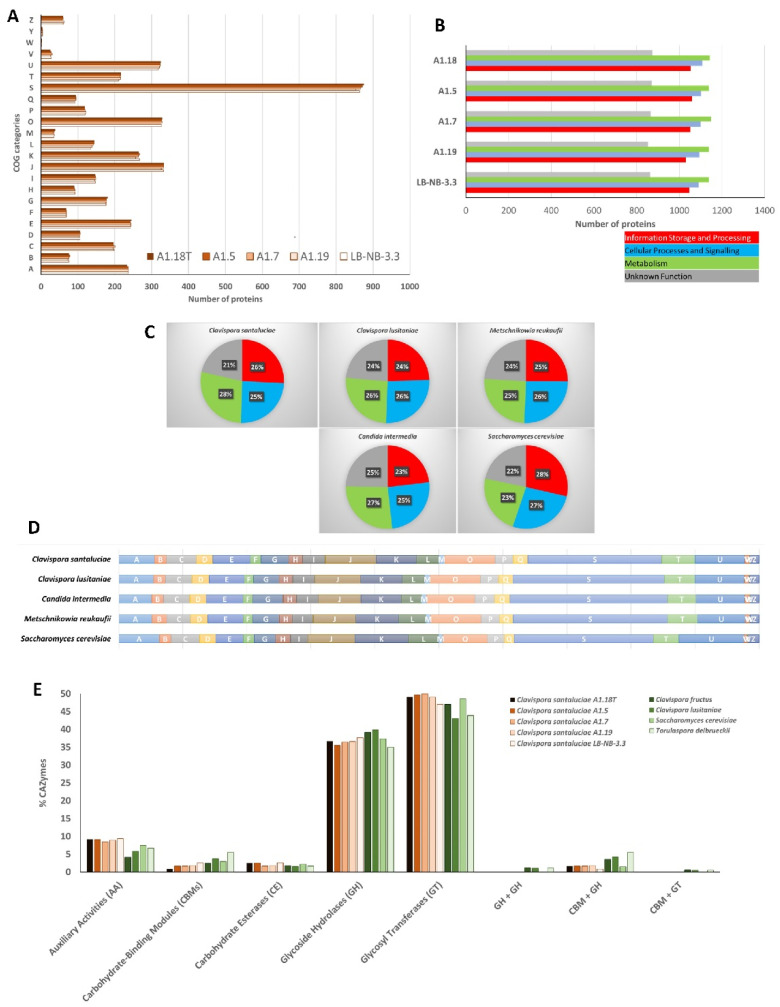
Functional annotation of *Clavispora santaluciae* genome: (**A**) proteome classification into 23 functional categories, corresponding to clusters of orthologous groups (COGs): A, RNA processing and modification; B, chromatin structure and dynamics; C, energy production and conversion; D, cell cycle control and mitosis; E, amino acid metabolism and transport; F, nucleotide metabolism and transport; G, carbohydrate metabolism and transport; H, coenzyme metabolism; I, lipid metabolism; J, translation; K, transcription; L, replication and repair; M, cell wall/membrane/envelop biogenesis; O, posttranslational modification, protein turnover, chaperone functions; P, inorganic ion transport and metabolism; Q, secondary Structure; S, function unknown; T, signal transduction; U, intracellular trafficking and secretion; Y, nuclear structure; Z, cytoskeleton; (**B**) classification of the annotated genes into four large functional categories; (**C**) comparison between the five *Clavispora santaluciae* strains and other relevant yeast species in proportions of the large functional categories; (**D**) comparison between relevant yeast species classification of the annotated genes into 23 COG categories; (**E**) percentage of CAZymes in the five sequenced genomes of *Clavispora santaluciae* and other relevant yeasts, showing the distribution of predicted proteins into major families.

For this analysis, eggNOG-mapper functionally annotated the predicted open reading frames of *Clavispora santaluciae*, providing important insights into their biological significance (Table 2, Figure 2). Between 3101 and 3103 genes were assigned to a KO category, corresponding to an average of 51.7% of all the annotated genes. A total of 4180 genes of *Clavispora santaluciae* type strain A1.18^T^ (68.6% of the total genes) were clustered into 24 COGs using eggNOG-mapper (Figure 2A), which were then classified into three main functional categories (Figure 2). This analysis revealed low variation between the five strains which is in accordance with the remaining annotation statistics shown before. Of note is the fact that the number of functionally annotated genes obtained in all strains varied between 68.5 and 69.1% (Table 2) and is rather low, as indicated by the high number of genes with “unknown function” in panel B of Figure 2 (gray bars; between 20.7 and 20.9%). However, these values are lower than those obtained for other species (Figure 2C, and category S in panel D), such as *Clavispora lusitaniae*, with 24%, (*Candida intermedia*), with 25%, and *Metschnikowia reukaufii*, with 24%, or even for *Saccharomyces cerevisiae* (22%) or *Torulaspora delbrueckii* (22%), as shown in our previous work [35]. This low number of genes with “unknown function” is a consequence of an improvement in the sequencing and annotation pipelines normally used to annotate yeast genomes.

Functional annotation of *Clavispora santaluciae* revealed that the highest percentage of annotated genes (Figure 2B) was related to “metabolism” (between 27.3 and 27.7%), followed by “cellular processes and signaling” (26.4–26.6%). This result is in agreement with that of other yeasts of Metschnikowiaceae (Figure 2, panels C and D), although this novel yeast species has a higher percentage of genes related to metabolism, which points to a superior biotechnological potential of this species. The importance of this value is even more evident if we compare it with the functional annotations of yeasts from other families, for which usually “information storage and processing” is the most represented category, as is the case of *T. delbrueckii* and *S. cerevisiae*, as previously shown [35]. The most abundant COG category in the genome of *Clavispora santaluciae* A1.18^T^ (panel A) was “translation, ribosomal structure, and biogenesis” (333 genes, representing 8% of the annotated genes), followed closely by “posttranslational modification, protein turnover, chaperones” (328/7.8%). The least abundant categories were “extracellular structures”, with only two associated genes.

Carbohydrate-active enzymes (CAZymes) were identified in the genome of *Clavispora santaluciae* by searching seven different families: auxiliary activities (AA), proteins containing a carbohydrate-binding module (CBMs), carbohydrate esterases (CE), glycoside hydrolases (GH), glycosyltransferases (GT), polysaccharide lyases (PL) and expansins (EXP), as well as combinations of the categories above (Figure 2E). Analysis of CAZymes revealed between 112 (strain A1.19) and 121 (strain A1.5) putative genes, distributed among five families, as no genes related with PL or EXP were detected. Approximately 1.97% of the total protein-coding genes in the *Clavispora santaluciae* genome encode CAZymes, which is in accordance with the reported range of 1 to 3% described for the generality of prokaryotes and eukaryotes [45].

In Figure 2E small inter-strain differences in *Clavispora santaluciae* CAZyome are noted, although strains A1.19 and LB-NB-3.3 have a slightly lower number of glycosyltransferases. The two classes with a higher number of annotated genes were glycosyltransferases and glycoside hydrolases. These CAZymes take part in the hydrolysis of glycosidic bonds between two or more carbohydrates or between a carbohydrate and a non-carbohydrate, as in the case of glycosyltransferases, or they assist in the formation of glycosidic bond and biosynthesis of polysaccharides, as in the case of glycosyltransferases. Interestingly, the CAZyome of *Clavispora santaluciae* reveals a control over complex carbohydrates, either being involved in their assembly (glycosyltransferases) or in their breakdown (glycoside hydrolases). To unravel genomic traits underlying the starch assimilative capacity [4], the presence of the enzymes GH31, GH13, GH57, and GH77 was analyzed since these are associated with an improved capacity to degrade starch [45]. Results (data not shown) revealed that *Clavispora santaluciae* CAZyome had genes *GH31* and *GH13*, which encode starch degrading enzymes, and their presence could explain the particular capacity of this species. However, closely related species of *Clavispora* and *Metschnikowia* branches revealed no differences in the annotation of these glycosyl hydrolases families [46], when compared with the novel species. In fact, despite differences in the total number of CAZymes, no particularly relevant differences were found between *Clavispora santaluciae* and other species of the same family, regarding important enzymes involved in cellulolytic, hemicellulolytic, and starch degradation (alignments and BLASTP results not shown). As *Clavispora santaluciae* was isolated solely from grapes, a comparison was performed between its CAZyome and that of the wine yeasts *S. cerevisiae* and *T. delbrueckii* (Figure 2E), selected for their involvement in the fermentation process and relevance in the winemaking industry [44,47]. Interestingly, all *Clavispora santaluciae* strains had a higher number of glycoside hydrolases and glycosyltransferases CAZymes than the other two wine yeasts, which points to great flexibility to both degrade or help synthesize complex compounds, which will lead to a release of glycosidically bound flavor compounds (such as terpenes and norisoprenoids) from naturally present grape glycosides and, therefore, most likely have a positive effect on wine mouthfeel and aroma [48,49].

Functional annotation of *Clavispora santaluciae* was also accomplished using KEGG Mapper—Reconstruct Pathway tool [32,34]. This tool completed KO-based mapping against KEGG databases, allowing us to visualize reconstructed global maps of metabolic pathways. Further, we used Reconstruct Pathway to evaluate pathway completeness for all five strains, together with the type strains of *Clavispora fructus*, *Clavispora lusitaniae,* and *Saccharomyces cerevisiae*. A total of 170 metabolic pathways were analyzed, and the results were categorized in a comparative heatmap (Supplementary Data S3). Reconstructed metabolic pathways of *Clavispora santaluciae* showed inter-strain differences, mostly in the strain LB-NB-3.3, in comparison with the remaining four, as a reflex of its isolation source. Most evident differences were detected in the citrate cycle, sphingosine degradation, threonine biosynthesis, and glutathione biosynthesis. Type strain A1.18^T^ showed marked differences from the other isolates, as it lacked some genes related to sulfate assimilation—namely, “assimilatory and dissimilatory sulfate reduction” (KEGG modules M00176 and M00596) and “sulfate–sulfur assimilation” (KEGG module M00616).

Comparison with other strains found in winemaking environments revealed similarity in the completeness of metabolic pathways (Supplementary Data S3), but with some important differences: (a) all *Clavispora santaluciae* strains lacked half the genes involved in tyrosine biosynthesis, in particular the ones responsible for the conversion of chorismite to tyrosine, as this pathway was complete in the other relevant wine yeast species; (b) KEGG modules M00013 (malonate semialdehyde pathway), M00143 (NADG dehydrogenase), M00066 (lactosylceramide biosynthesis), M00546 (purine degradation), M00133 (polyamine biosynthesis), and M00793 (rhamnose biosynthesis) revealed higher level of completeness in yeasts from Metschnikowiaceae than in *S. cerevisiae*; (c) module for Leucine degradation revealed higher completeness of metabolic pathways in *Clavispora santaluciae*, compared with *Clavispora lusitaniae*.

### 3.4. Interspecific Genomic Variability of Metschnikowiaceae

**Figure 3:**
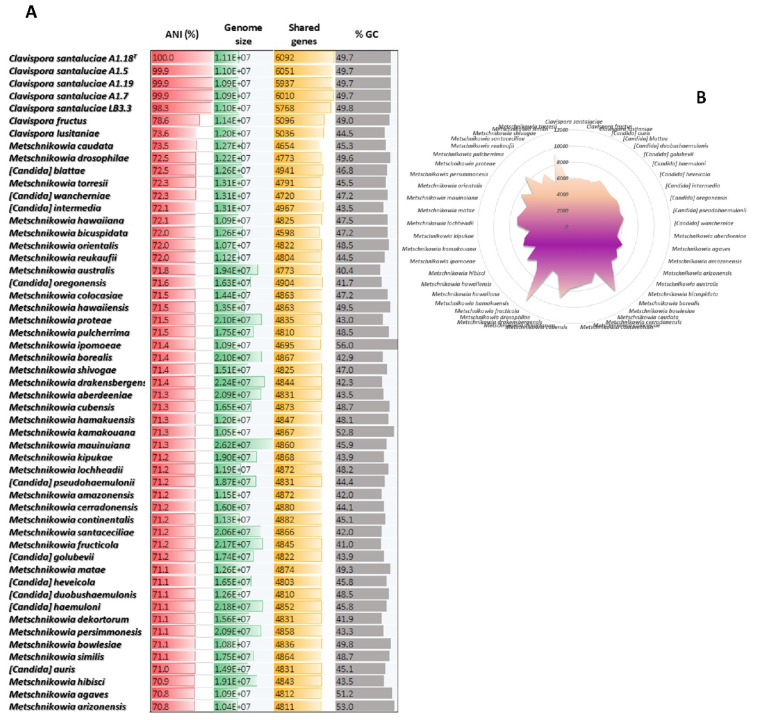
Comparative genomics of Metschnikowiaceae yeasts: (**A**) average nucleotide identity (% ANI), genome size (Mbp), number of coding genes, and percentage of GC among the complete genomes of Metschnikowiaceae species; (**B**) number of coding genes across the Metschnikowiaceae family.

Comparative genomics between *Clavispora santaluciae* and the other species of the Metschnikowiaceae was evaluated using the pairwise average nucleotide identity values (ANI in %), genome size, number of shared genes using type strain A1.18^T^ as query, and percentage of guanine–cytosine bases (GC) in the genome sequences. Figure 3 shows that the number of homologous coding genes is not correlated with the genome size of Metschnikowiaceae. As sequencing of ribosomal DNA regions has shown [4], *Clavispora santaluciae* is the closest relative to *Clavispora fructus* and *Clavispora lusitaniae*, with the ANI similarity of 78.6% and 73.6%, respectively. Although all the genomes in the present work were reannotated, using the same pipeline in order to avoid incongruences, care must be taken when analyzing genome sizes, since different sequencing technologies and assembly approaches were used by the different authors.

The haploid genome of *Clavispora santaluciae* is interestingly small (average size of 10.9Mb), in contrast to the average 15.3 Mb of the other species of this family (Figure 3A). This small genome size is also reflected in the lower number of protein-coding genes (6049) identified on average for the five *Clavispora santaluciae* which is similar to the average number (5972) determined in the other *Clavispora* species but significantly lower than for the other species of Metschnikowiaceae (on average 6744; Figure 3B). An extreme example is *Metschnikowia fructicola*, with more than 10,000 genes revealed by our genome annotation pipeline, which is a significant increase, compared with previous genome annotation (8629) of this species [46]. From Figure 3 we can observe a large diversity between species of *Metschnikowia* in terms of their genome size (panel A) and the number of coding genes (panel B), while *Clavispora/*(*Candida*) strains generally have smaller genome sizes and fewer predicted proteins. This fact could be related to evolutionary constraints, as species of *Metschnikowia* are usually associated with diverse environments and substrates, while *Clavispora/*(*Candida*) yeasts are typically associated with a few habitats. In particular, *Clavispora santaluciae* yeasts were only isolated from grapes until now. The smaller genome size and its lower number of coding genes could be related to a lower capability to adapt to new environments. Although a direct link between small genome size and evolutionary plasticity has not yet been established, to our knowledge, some reports link these features in recent years. For example, Steenwyk et al. in 2019 [50] showed that yeasts of the genus *Hanseniaspora* benefit from their reduced genome sizes by the ability to grow rapidly. They showed that the two *Hanseniaspora* lineages exhibit very high evolutionary rates and that the lineage had lost many of the genes involved in cell cycle and DNA repair mechanisms during evolution, and has, therefore, been able to diversify more rapidly. On the other hand, other studies show that yeasts with larges genomic sequences have redundant genomes, linked to a strong tendency for map dispersion, visible by duplication of non-coding RNAs, the spread of tRNA genes, and a high number of tRNA genes, as is the case of *Yarrowia lipolytica* [51]. One last example refers to the evolution of Saccharomycotina yeasts [52], for which it was shown that genes seem to be very rarely gained by horizontal gene transfer, while gene losses are more common, along with the loss of whole sets of genes in some pathways in some species.

### 3.5. Phylogenomics of Metschnikowiacea

**Figure 4:**
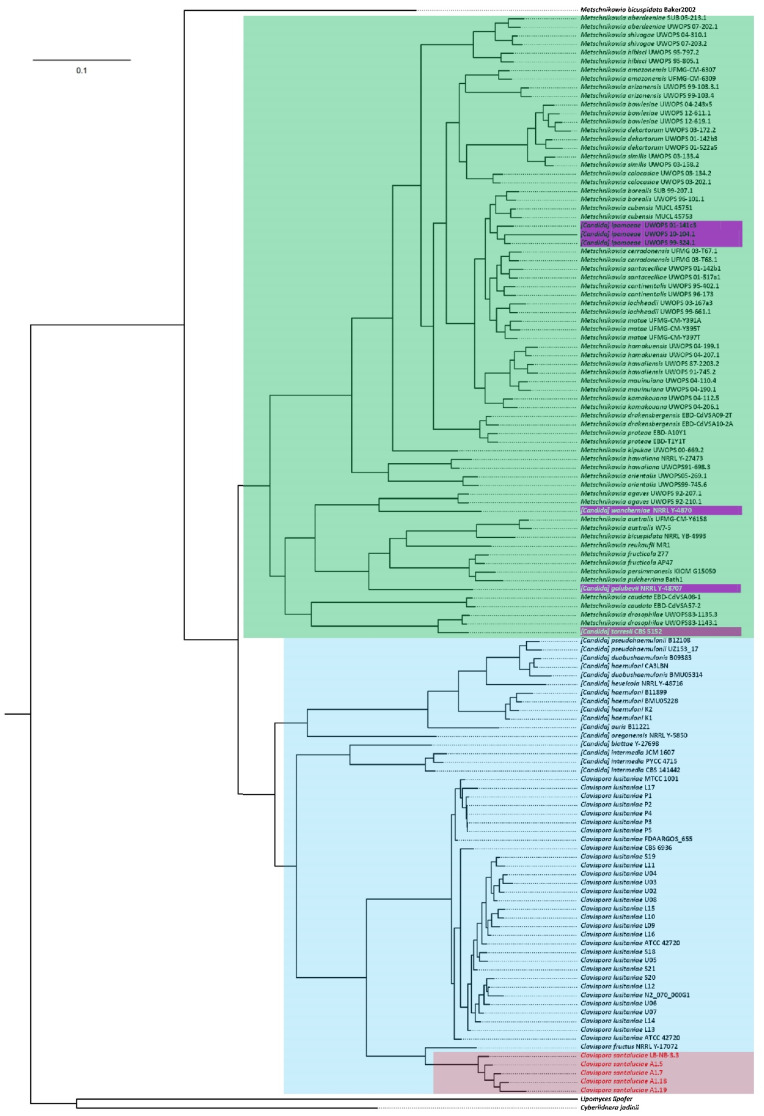
Phylogram of Metschnikowiaceae family showing the core proteome of 126 yeast strains (alignment of 2362 common proteins). *Clavispora santaluciae* genomes, sequenced in the present work, are highlighted using a red box, while *Clavispora/Candida* and *Metschnikowia* genera are highlighted by blue and green boxes, respectively. Incongruent locations are highlighted by purple boxes. Phylogenetic reconstruction was performed by considering maximum likelihood and 500 bootstrap replicates of the concatenated alignments. Bootstrap values were omitted as they were 100% for all branches.

The phylogenomics of Metschnikowiaceae was determined for all 121 strains (48 species) with complete genomes available, as well as the 5 *Clavispora santaluciae* whose genomes were sequenced and assembled in the present work. The complete proteome of the type strain A1.18^T^ (6092 proteins) was used in a BLASTP analysis against the Metschnikowiaceae proteomic database (with two outgroups), composed in the current study, and a total of 2362 proteins had homologs in the 126 yeasts. The phylogenetic tree highlighted in Figure 4 represents the alignment of the core concatenated proteins. This phylogram represents the most comprehensive phylogenetic assessment of Metschnikowiaceae in which complete genomes were analyzed. As expected, the five *Clavispora santaluciae* strains formed a homologous clade (highlighted in red in Figure 4), separated from *Clavispora fructus*, and from strains of *Clavispora lusitaniae*. The phylogenetic distributions observed with our genome analysis generally agree with the taxonomic phylogeny shown before [4], using alignments of the ITS and D1/D2 regions, with minor exceptions. In the complete-genome analysis, strains A1.18^T^ and A1.19 were revealed to be closest relatives and separated from strains A1.7 and A1.5. The strain LB-NB-3.3 isolated from Italy was most distantly related. However, the phylogeny of ITS and D1/D2 regions showed the highest similarity between strains A1.18^T^ and A1.5. This separation between later strains, when analyzing complete genomes, can also be observed in the dot plots of Figure 1, and stresses the need for complete-genome analysis to establish robust phylogenies. Species of (*Candida*) were grouped on a common clade, separated from the “true” *Clavispora* yeasts (blue box, Figure 4), with the exception of (*Candida*) *golubevii* and (*Candida*) *wancherniae*, which are highlighted by pink boxes in Figure 4.

Bootstrap values of 100% were found for all branches, confirming the robustness of the phylogram. Monophyletic branches were obtained for all species with more than one strain, with few exceptions: (a) strain *M. dekortorum* UWOPS 03-172.2 clustered together with the two strains of *M. bowlesiae*, and separately from the other two strains of *M. dekortorum*; (b) one of the three strains of (*Candida*) *haemuloni*—CA3LBN—clustered in a monophyletic branch with (*Candida*) *duobushaemulonis,* far from other strains of the same species; (c) strain *M. bicuspidata* Baker2002 was placed outside the main group of Metschnikowiaceae strains, serving as an outgroup of this larger group and showing clear differences from the strain *M. bicuspidata* NRRL YB-4993. This last observation needs careful validation because, in the work of Lachance et al. [13], *M. bicuspidata* NRRL YB-4993 was used as an outgroup in the established phylogeny of *Metschnikowia,* to root the tree, under the justification of being divergent from the remaining large-spore species. However, in the current work, in addition to the large difference detected, in terms of genomic contents, between both available genomes of *M. bicuspidata*, a similarity was also observed between type strain NRRL YB-4993 and *M. australis* and *M. reukaufii*. In addition, in the work of Shen et al. [14], *M. bicuspidata* was also placed outside the main *Metschnikowia* species group, clustered with the type strain of *Candida golubevii*. This fact points to the importance to include different strains in phylogenetic analysis to clarify species positioning. In the future, additional *M. bicuspidata* strains should be sequenced and included in a phylogenomic analysis in order to clarify the position of this species.

The two other incongruent (*Candida*) inclusions described above as having bootstrap support of 100% in the phylogram of Figure 4—namely, *(Candida*) golubevii and (*Candida*) wancherniae, represent clear candidate species whose nomenclature needs to be revised and included in the *Metschnikowia* genus. These species had also particular placements in the phylogeny established by Shen et al. [14], presenting an intermediate position between (*Candida*) and *Metschnikowia* genera.

## 4. Conclusions

*Clavispora santaluciae* is a novel non-*Saccharomyces* yeast species, recently isolated from grapes of Azores vineyards, a Portuguese archipelago with particular environmental conditions, and from Italian grapes infected with *Drosophila suzukii*. In the current work, complete genomes of all the described *Clavispora santaluciae* strains were sequenced, assembled, and annotated. With this work, we increase the number of Metschnikowiaceae yeasts with the sequenced and annotated genome. By using a combination of long- and short-read sequencing technologies to sequence strains´ genomes, we were able to obtain high-quality and complete DNA sequences, which allowed us to predict a high number of coding sequences and robust sequencing statistics. This high number of protein-coding genes might not be related to any particularity of this yeast´s genome but rather a consequence of an improvement of the sequencing technology and the annotation pipeline.

Genome comparison revealed particular differences between strains of *Clavispora santaluciae*, reflecting their isolation from two different ecological niches—Azorean and Italian vineyards—as well as mechanisms of adaptation to the intricate and arduous environment features of the geographical location from which they were isolated. In particular, the differences in terms of number of coding genes (shared and unique), number of the transposable elements, the amount and diversity of non-coding RNAs, and enzymatic potential of each strain through CAZyome analysis were detailed in the present work to unravel mechanisms of adaptation to both environments. These differences, primarily the ones found between Italian and Portuguese strains, echoes mainly climatic differences in the strains´ origin. While Italian grapes were obtained in vineyards from the variety Vernatsch, for which the influential climatic condition is the warm, moist, and continental weather, grapes from Azorean vineyards are subject to a particular and aggressive microclimate due to the basaltic stone soils. Results show different adaptation mechanisms underlying the occurrence of these yeasts in nature, as, for example, the absence of transposable elements in the strain isolated from Italy, which was sufficient to leave a marked fingerprint in their genomes.

A future increase in the number of *Clavispora santaluciae* strains will allow the use of populational analysis methods to unravel new mechanisms of adaptation to the environment and to explore new practical applications of these isolates [53,54,55,56]. In detail, genome-wide environmental associations could be explored recurring to algorithms described for targeted mapping [57], linkage disequilibrium (LD) measures could allow the dissection of genetic diversity [58], and new approaches based on machine learning algorithms can open new doors to discover novel biotechnological applications [59,60].

Comparison of *Clavispora santaluciae* with other yeast species successfully unraveled the presence of distinct traits that elevate this species potential for biotechnological applications. The small genome size combines a high number of protein-coding genes and a high percentage of metabolic pathways completeness. In its CAZyome, *Clavispora santaluciae* revealed a high number of glycoside hydrolases and glycosyltransferases, even higher than the ones existing in traditionally used wine yeasts. This discovery reflects great flexibility to both degrade or synthesize complex compounds, both with potential interest in winemaking and in other biotechnological industries.

Using complete genomes of Metschnikowiaceae, we presented the largest ever phylogenetic assessment of this yeast family, highlighting particular differences to other phylograms with less robustness that use only some parts of the ribosomal genes. With this analysis, it was possible to identify three (*Candida*) species whose nomenclature needs to be revised. The growing knowledge about this yeast family unravels new potential applications of these species as the high genomic plasticity may also correlate to a larger phenotypic diversity and a higher propensity to adapt to new environments.

## Figures and Tables

**Table 1:** Genome assembly statistics of *Clavispora santaluciae* strains.

		A1.18^T^	A1.5	A1.7	A1.19	LB-NB-3.3
Canu assembler	Assembly length (bp)	11,088,431	11,018,248	10,921,443	10,861,576	11,019,028
	Number of scaffolds	43	13	46	86	30
	N50 (bp)	315,943	802,369	355,153	1,329,122	494,470
	L50	7	3	6	14	7
	Number of N’s per 100 Kb	0	0	0	0	0
	Number of scaffolds > 5000 bp	29	11	28	53	23
	Total length > 5000 bp	10,780,215	10,974,595	10,557,056	10,118,395	10,856,688
Masurca assembler	Substitution errors revised	42	8	141	428	70
	Insertion/Deletion errors revised	1686	536	2825	6144	1607
	Assembly length (bp)	11,089,145	11,018,616	10,922,446	10,863,639	11,019,715
	Number of scaffolds	43	13	46	86	30
	N50 (bp)	532,329	1,048,728	654,799	218,696	650,701
	L50	7	3	6	14	7
	Number of N’s per 100 Kb	0	0	0	0	0
	Number of scaffolds >5000 bp	29	11	28	53	23
	Total length >5000 bp	10,780,920	10,974,959	10,558,055	10,120,373	10,857,358
RagTag assembler	Assembly length (bp)	11,092,545	11,019,016	10,925,846	10,870,339	11,021,815
	Number of scaffolds/chromosomes	**9**	**9**	12	19	**9**
	Number of N´s per 100 Kb	3065	3.63	31.12	61.64	19.05
	Number of scaffolds >5000 bp	4	8	4	3	7
	Total length >5000 bp	11,025,073	11,000,234	10,766,609	10,606,285	10,966,127
Ploidy		haploid	haploid	haploid	haploid	haploid
GC content (%)		49.66	49.70	49.73	49.66	49.76

**Table 2:** *Clavispora santaluciae* genome annotation statistics.

	A1.18^T^	A1.5	A1.7	A1.19	LB-NB-3.3
**Protein coding genes**					
Total number	6092	6034	6067	6015	6038
Range of protein lengths (aa)	66–4974	63–4974	57–4974	60–4974	66–5293
Average protein length (aa)	557.6	556.6	550.9	543.5	518.3
**Non-coding RNAs**					
microRNAs (miRNAs)	32	32	33	31	21
small RNAs (sRNA)	20	21	22	20	23
nuclear RNAs (snRNA)	7	7	6	7	7
nucleolar RNAs (snoRNA)	93	91	99	94	98
long noncoding RNAs (lncRNA)	8	8	9	8	12
ribosomal RNAs (rRNA)	96	63	42	69	124
transfer RNAs (tRNA)	276	259	279	299	248
Other	29	32	32	35	32
**BUSCO Orthologs** *Ascomycota odb10 database*					
Genome Completeness (%)	93.5	94.4	93.4	90.7	93.6
Complete BUSCOs	1595	1611	1594	1547	1597
Fragmented BUSCOs	17	14	18	21	4
Missing BUSCOs	94	81	94	138	94
*Saccharomycetes odb10 database*					
Genome Completeness (%)	98.0	99.1	98.0	95.1	98.2
Complete BUSCOs	2094	2118	2094	2032	2099
Fragmented BUSCOs	14	11	12	17	13
Missing BUSCOs	29	8	31	88	25
**Eggnog-mapper functional annotation**					
Genes with KO assigned	3130 (51.4%)	3129 (51.9%)	3125 (51.6%)	3130 (52.0%)	3101 (51.4%)
Genes with COG assigned	4180 (68.6%)	4171 (69.1%)	4166 (68.7%)	4119 (68.5%)	4141 (68.6%)
**CAZymes functional annotation**					
Number of genes annotated	120	121	118	112	117

## Data Availability

The sequencing data are available at NCBI BioProject ID PRJNA784374.

## Supplementary Materials

The following are available online at https://www.mdpi.com/article/10.3390/jof8010052/s1. Supplementary Data S1: *Clavispora santaluciae* genome annotations, Supplementary Data S2: non-coding RNAs predicted in the genome of *Clavispora santaluciae*, Supplementary Data S3: Heatmap representing pathway completeness in *Clavispora santaluciae*, *Clavispora lusitaniae*, *Clavispora fructus*, and *Saccharomyces cerevisiae*. Completeness values were obtained using KEGG Mapper—Reconstruct tool, and organized using KEGG Modules information.
